# Why Older Adults Recall Autobiographical Memories From Their Youth

**DOI:** 10.1093/geroni/igaa057.1184

**Published:** 2020-12-16

**Authors:** Tabea Wolf, Daniel Zimprich

**Affiliations:** Ulm University, Ulm, Baden-Wurttemberg, Germany

## Abstract

When older adults are asked to remember their lives, they recall disproportionally more events from their youth (e.g., Rubin, Rahhal, & Poon, 1998). This phenomenon, called the reminiscence bump, is one of the most robust findings in autobiographical memory research. Whereas most explanatory accounts have focused on differential encoding and retention of memories experienced during one’s youth (e.g., Rubin et al., 1998), recent research also puts emphasis on the retrieval of memories (e.g., Glück & Bluck, 2009; Rubin & Berntsen, 2003). In the present study, we take a functional perspective on the reminiscence bump and examine why older adults recall memories from their past. Participants (age 57-89; N = 112) reported memories in response to 30 emotionally neutral cue-words and self-rated each memory for serving directive, social-bonding, self-continuity, and mood-enhancing functions (Wolf & Demiray, 2019). The age distribution shows an early reminiscence bump located between the ages of 6 and 20 years. Compared to memories from later life periods, memories from the reminiscence bump more frequently serve self-continuity and less frequently directive and mood-enhancing functions. No differences were found regarding the use of memories for social-bonding. The results strengthen the assumption that experiences from one’s youth serve to maintain a sense of self-continuity throughout the lifespan (e.g., Rathbone et al., 2008). To cope with current problems or emotions, however, older adults are more likely to draw on experiences from their adult life – probably because these experiences are more similar to what they are experiencing now.

